# Localised Œdema

**Published:** 1895-02-16

**Authors:** G. Parker

**Affiliations:** Assistant Physician, Bristol General Hospital


### MEDICAL PROGRESS AND HOSPITAL CLINICS.

[*The Editor will be glad to receive offers of co-operation and contributions from members of the profession. All letters should be addressed to* THE EDITOR, THE LODGE, PORCHESTER SQUARE, LONDON, W.]

### LOCALISED ŒDEMA.

By G. PARKER, M.A., M.D., Assistant Physician, Bristol General Hospital.

A WOMAN with irregular swellings on her face, aged fifty, who proved to be a well marked case of Quincke's angio-neurotic œdema, recently came to me as an out-patient. Instances of this affection are somewhat rare, and it may be useful to compare it with other forms of localised œdema.

The etiology of these cases of localised œdema is obscure, but something may be learnt by comparison with such forms as common urticaria, phlegmasia dolens, elephantiasis, and the œdemas arising on the cheek in disease of the antrum, over the ribs in empyœma, and around joints and other areas affected by rheumatism. Moreover, some light may be thrown on the production of general œdema by the consideration of these simple forms, since in them we can exclude the possibility of any general alteration of the blood, which has been adduced by one school of writers as an element in the production of general œdema. There is, indeed, much evidence to show that in every case the real change is in the vessel walls, or in the forces of the circulation; but we may exclude the possibility of error if we concentrate our attention on some simple type of localised œdema, such as the wheals of urticaria, where it is clear that no alteration in the general stock of blood has taken place. Unna and others divide all œdemas into inflammatory and mechanical. Unna himself is remarkable for the persistency with which he refers almost every variety to the latter type. They are caused, he argues, by venous obstruction, or a disproportion between the tonus of the veins and arteries. Closure of all the lymphatic channels of a limb does not give rise to œdema in it, while obstruction of the veins never fails to produce it. The lymphatics normally only convey that small fraction of lymph which does not return by the venous capillaries. Moreover, from the possibility of injecting the lymphatics direct from the capillaries, it is argued that the free anastomoses between the two systems would cause merely regurgitation into the venous capillaries if the lymphatics were obstructed. In occlusion of the thoracic duct, indeed, if collateral channels are not opened, the veins do not take up the whole of the fluid; but in peripheral vessels, at any rate, the claim is made that lymphatic occlusion does not cause, though it may increase, an existing œdema.

In inflammatory œdema, though the production of the œdema depends on specific changes in the vessel walls, the fluid would be immediately carried off, unless there were either a difficulty in its re-entering the vessels or venous or lymphatic obstruction. The former is probably the true explanation, though possibly the increased amount of lymph overtaxes the small carrying power of the lymphatics.

Unna explains urticarias as spastic œdemas caused by a sudden contraction of the walls of the venules, but why this is greater than the contraction of the arteries is not, in my opinion, at all clear. He finds, too, that if a tight bandage be placed round a limb affected with nettle-rash the wheals become flattened and depressed, and the itching ceases even before the general swelling and mechanical œdema become visible, that is, "the increased pressure on the larger veins of the skin overcomes the spasm of the smaller veins." It would seem, however, quite reasonable to suppose that a venous œdema caused by the bandage has really been added to and hides an existing sympathetic one. Still the observation is an important one. In the sudden urticarias we may remark the tense, elastic, sharply defined swelling while the normal elasticity of the skin is still perfect, as contrasted with the loose, irregular, easily pitting œdema which occurs in more chronic affections where the relaxed tissues permit a ready movement of lymph. Thus it becomes possible, if indeed the theory of spastic œdemas is a reality, to group with urticaria, the œdema of rheumatism, that of various poisons which affect the tone of the vessels through the nerves, and even the œdema of paralysed limbs, where it is supposed that among the vaso-motor changes which have occurred the contraction of the venules exceeds that of the arterioles.

There is, however, much to be said in favour of regarding these varieties as simply produced by various lesions and changes in the vessel walls. The evidence of venous spasm is too slight, and the analogy to true inflammatory œdema too close for us to readily accept Unna's view. Indeed the tendency is to regard inflammation itself and the accompanying diapedesis as a toxic process affecting the vessel walls. If this same open condition of the walls continues, as possibly in a paralysed limb, the difficulty of getting rid of the fluid may account even for chronic œdemas. Kaposi, as the explanation of the angio-neurotic œdemas, believes in a primary contraction of the arterioles and then a period of dilatation, with slowing of the blood current, followed by effusion, not necessarily inflammatory in character, though how an increased permeability of the vessel wall is brought about by vaso-motor changes is not too clear. It is noticeable that the distribution of urticaria is irregular, and not confined to the course of certain nerves like zona. The giant urticaria, which comes and disappears rapidly, retains the defined form of the common variety; but Quincke's œdema, which slowly diminishes in parts while fresh patches swell up, is soft and irregular in shape. My patient has suffered for years from these unsightly patches under the eyes, along the lower lip, and on the cheek. The face is rarely quite free, and the eyes are at times nearly closed. The skin is normal in appearance, and there is neither pain nor itching, though the swellings are an inch or two across, and quite an inch high. The general health is good, the urine free from albumen, and the heart normal. Two or more joints in each hand are marked by the lesions of rheumatoid arthritis, a disease which, according to Jamieson, is in some connection with this œdema. He reports similar cases, one of which began with pain over the left orbit. The œdema here spread to the larynx, which constitutes the chief danger of the affection. Two deaths from this cause are mentioned by Osler. As to the ætiology very little is known. Some patients appear to suffer before the attacks from gastro-intestinal disturbances and severe colic, and the complaint is apt to be hereditary. Thus twenty-two members of one family were found to be sufferers from it. D. Wills and Cooper, in reporting five recent cases in "Brain," consider that it has often to do with the onset of puberty or the menopanse. Though, however, the occasional occurrence of digestive troubles, its itching and heat, its sudden appearance and irregular distribution, connect it with common urticaria, nothing definitely is known of its origin. Unna indeed supposes that a spasm is brought about in a vein supplying a larger area than in common urticaria, but of this there is no proof.

To turn to other types, the pathological lesion in phlegmasia dolens is probably thrombosis in the uterine veins, spreading to those of the thigh. The absence of pitting depends on the richness of the lymph in albumen at the time of parturition or upon some septic inflammation. A whole class of œdemas arise in the same way. Thus an elderly woman came to me some time ago with repeated attacks of so-called erysipelas in the right ear. This remains disfigured by a brawny thickening from the organised lymph, which was poured out during the attacks. I have long had under observation two patients, one of whom shows a well-marked instance of elephantiasis nostras affecting one leg and the other has a pair of legs resembling bolsters tightly stuffed with straw. In both cases there have been recurrent inflammatory attacks, and the second one has some heart weakness in addition. The œdema is brawny, pitting very little, and in the first case the skin is forced outwards into huge irregular protuberances and folds, containing a more or less solid material bound down by adhesions and bands, very different from the uniform fluid distension seen in recent dropsy. Whether the inflammatory attacks seen in such cases are true erysipelas is very doubtful. Pleural and other effusions easily change from a serous to a coagulative type under septic influences of various kinds, and it is not certain that erysipelas is due only to the streptococcus erysipelatis.

Unna combats the view that the tropical elephantiasis is due to plugging of the lymphatics, and points out that filiaria embryoes are certainly found also in the blood vessels, and that any obstruction in the lymphatics is of secondary importance. Still it is difficult to see why this should not produce ordinary fluid œdema. Cures have indeed been reported after ligature of the fomeral artery. Perforation of the lymphatics doubtless occurs, but I think that here again some septic coagulation or inflammation must exist. Another form of localised œdema is seen over the ribs in empyæma, and on the cheek in disease of the antrum. These and the œdema which extends round an abscess outside the inflamed area are regarded by Unna as mechanically caused by the excessive flow of blood from the inflamed area, but many hold that they are instances of true inflammatory œdema. Of the latter type we need say little; the change in the vessel walls, the diapedesis, and the chemiotaxic processes are well known, and for their discussion we have not space here. The question is rather how far may the mechanical œdemas be considered as really inflammatory and caused by changes in the vessel walls? Does obstruction act simply by causing filtration, or does it produce alterations in the vessel walls? Does a blow produce spasm of the vessels or increased permeability? Are the vessels of a paralysed limb under local vasomotor spasm, or are the walls unhealthily porous? On the whole, it would seem that the class of mechanical œdemas is smaller than appears at first sight and that the process is more frequently an active effusion than a mere leakage.

